# Treatment failure and associated factors among first line patients on highly active antiretroviral therapy in Ethiopia: a systematic review and meta-analysis

**DOI:** 10.1186/s41256-019-0120-4

**Published:** 2019-10-30

**Authors:** Moges Agazhe Assemie, Muluneh Alene, Daniel Bekele Ketema, Selishi Mulatu

**Affiliations:** 1 Department of Public Health, College of Health Science Debre Markos University, P.O. Box 269, Debre Markos, Ethiopia; 2 Department of Nursing, School of Health Science Bahir Dar University, Bahir Dar, Ethiopia

**Keywords:** Antiretroviral therapy, First-line treatment, Treatment failure, Switching to second-line, HIV/AIDS, Ethiopia

## Abstract

**Background:**

Antiretroviral therapy (ART) restores immune function and reduces human immunodeficiency virus (HIV) related adverse outcomes. The results of previous studies in Ethiopia were replete with inconsistent findings; nonexistence of national representative figures and determinant factors are found as significant gap. The aim of this systematic review and meta-analysis was to assess the existing evidence on ART treatment failure and associated factors in Ethiopia.

**Methods:**

Relevant studies on ART treatment failure were retrieved from international databases: PubMed, Google Scholar, Scopus, and Science Direct systematically prior to March 14, 2019. All identified studies reporting the proportion of first line treatment failure among HIV patients in Ethiopia were included. Two authors independently extracted all necessary data using a standardized data extraction format. A random-effects model was used to calculate pooled estimates and associated factors in Stata/se Version-14. The Cochrane Q test statistics and *I*^*2*^ tests were used to assess the heterogeneity of the studies.

**Results:**

From 18 articles reviewed; the pooled proportion of first line treatment failure among ART users in Ethiopia was 15.3% (95% CI: 12, 18.6) with (*I*^2^ = 97.9%, *p* < 0.001). The subgroup analysis by World Health Organization (WHO) treatment failure assessment criteria were carried out, accordingly the highest prevalence (11.5%) was noted on immunological and the lowest (5.8%) was observed virological treatment failure. We had found poor adherence (OR = 8.6, 95% CI: 5.6, 13.4), not disclosed (OR = 2.1, 95% CI: 1.5, 3.0), advanced WHO clinical stage III/IV (OR = 2.4, 95% CI: 1.5, 3.8), change in regimen (OR = 2.5, 95% CI: 1.6, 3.9) and being co-infected (OR = 2.56, 95% CI: 2.2, 3.0) were statistically significant factors for treatment failure.

**Conclusion:**

In this study, treatment failure among ART users in Ethiopia was significant. Adherence, co-infection, advanced WHO clinical stage, regimen change, and disclosure are determinant factors for treatment failure. Therefore, improve drug adherence, prevent co-infection, close follow up, and prevent HIV-drug resistance are required in future remedial efforts.

## Background

Ethiopia is one of the low-income countries experiencing high communicable disease burden, including HIV/AIDS which accounts 70 disability adjusted life years per 100,000 individuals [1, 2]. Access to highly active antiretroviral therapy (HAART) in Ethiopia started in 2005, and reached 420,000 people from 716, 418 people living with HIV/AIDS by 2016 ART [3, 4]. Even though ART is not a curative medicine, access to HAART has played a vital role in the clinical management of HIV- infected individuals through reestablishing the immune function and preventing morbidity and mortality. HAART also expected to contribute significant role to reduce new HIV-infection by 2020 [5, 6].

Even though many HIV-positive clients have accessed ART, first line treatment failure continues to grow in resource limiting countries. First-line antiretroviral treatment is a combination of two nucleoside reverse-transcriptase inhibitors plus a non-nucleoside reverse-transcriptase inhibitor while treatment failure is the progression of HIV infection after the initiation of ART [7, 8].

Failure can be assessed, based on WHO criteria, as clinical, immunologic, virologic or a combination. Regular treatment failure detection is low because of inadequate capacity and lack of laboratory facilities in resource-limited settings including Ethiopia [9].

Treatment failure is frequently linked to mortality, which is costly locally, and the development of drug resistant viral strains, which has global implications [10, 11]. The imperative for compliance with lifelong use of medication to avert negative outcomes is a significant challenge [12–14]. Therefore, early detection of treatment failure is a key to sustain first-line therapy effectiveness and to prevent HIV-drug resistance [12, 13].

There are many studies conducted in Ethiopia to determine the prevalence of first line treatment failure [15–31]. However, the magnitudes of these studies were inconsistent and characterized by great variability: nonexistence of country wide data to represent national treatment failure and its determinant factors are identified as significant gap. The aim of this systematic review and meta-analysis was to estimate the pooled prevalence and associated factors of treatment failure in the Ethiopia. This finding will assist decision makers and other concerned stakeholders to design, implement and evaluate effective and efficient interventions to improve the ART adherence in order to decrease morbidity, mortality, and development of drug resistance.

## Methods

### Study design and settings

Systematic review and meta-analysis using computerized databases; searches were performed to locate all studies to estimate the pooled prevalence of treatment failure in HIV/AIDS patients in Ethiopia which is found in east Africa.

### Search strategy

To find potentially relevant articles, a comprehensive search was carried out on Pub Med/MEDLINE, Google Scholar, Scopus and Science Direct databases. We extended our search by retrieving and extracting potential articles from reference lists of eligible articles as well as hand searching for grey literature and other relevant literature collections. The search of the literature was conducted between April 2018 and March 2019. All papers published prior to March 14, 2019 were considered. The search protocol was formulated by using common key words ‘prevalence OR magnitude AND associated factor OR first-line OR switching OR second-line OR treatment failure AND antiretroviral therapy OR HIV/AIDS OR resource-limited setting AND ‘Ethiopia’. We followed and presented this meta-analysis according to the Preferred Reporting Items for Systematic Reviews and Meta-Analysis (PRISMA) guidelines [32].

### Eligibility criteria

#### Inclusion criteria

##### Study area

All studies conducted in Ethiopia were included in the systematic review and a combination of two and above studies used in the meta-analysis to produce single estimate of common effect.

##### Study design

All observational study designs reporting the prevalence of first line treatment failure.

##### Population

All HIV positive patients on first line antiretroviral treatment.

##### Language

Only articles reported in English language.

##### Publication condition

Published and unpublished articles.

##### Exclusion criteria

Articles, which were not fully accessed or when they are not fully explain first line treatment failure. Exclusion of these articles is because of the inability to assess the quality of articles in the absence of full text.

### Outcome variables

This study has an outcome variable prevalence of first line treatment failure, which is defined as clinical, immunological, virological or some combination of those outcomes [7] and determinant factors. We determine the association between treatment failure and associated factors in the form of the log odds ratio. The determinants included in this review were adherence, advanced WHO stage, CD4 count, regimen change, functional status, co-infection and disclosure.

### Data extraction

All identified studies were screened via title and abstract for inclusion by two reviewers (MA and DBK) independently extracting all necessary data. Discussions and mutual consensus processes were undertaken when disagreements were raised between the two reviewers. These reviewers then assessed the full text of potentially eligible papers. The primary author of the original research was contacted for additional information or to clarify method details as needed. The data extraction format included primary author, publication year, regions of the country, study area, sample size and prevalence with 95% CI.

### Quality appraisal

The included articles were evaluated for quality, with only high-quality studies included in the analysis. Two authors (MAA and MA) independently assessed the quality of each included paper using an adapted Newcastle-Ottawa Scale quality assessment tool for cross-sectional and retrospective cohort studies [33]. The tool has three sections in general; the first section is graded out of five stars and considers the methodological quality of each original study. The second section deals with the comparability of the study cases or cohorts, with a possibility of two stars to be gained. The third section focus on the outcome and statistical analysis of each original study with a possibility of three stars to be given. The reviewers compared their quality appraisal scores and resolved any discrepancy before calculating the final appraisal score. Articles with a score of ≥6 out of 10 scales were considered high quality, which, in this instance, reflected all eligible studies (i.e., none were eliminated at this stage).

### Data processing and analysis

Data on study design, sample size, study population/age group, mid-study period and publication year were extracted in Microsoft Excel format, and then analysis was carried out using STATA/se Version 14 software. Heterogeneity among reported prevalence was assessed by computing *p*-values of Cochrane Q-test and *I*^2^-statics [34]. The pooled prevalence of treatment failure was carried out with a random effects meta-analysis model, generating the pooled 95% confidence interval using the DerSimonian and Laird’s method [35]. Sub group analysis was done to investigate how treatment failure varies across different sub-group of patients and to minimize the random variations between the point estimates of the primary study; subgroup analysis was done based on WHO treatment failure assessment criteria, study design and sample size. In addition, univariate meta-regression was undertaken to identify the possible source of heterogeneity. Publication bias was assessed by Egger’s and Beggs’ tests at 5% significant level [36]. Point prevalence, as well as 95% confidence intervals, was presented in the forest plot format. In this plot, the size of each box indicated the weight of the study, while each crossed line refers to 95% confidence interval. For the second outcome, a log odds ratio was used to examine the association between determinant factors and treatment failure.

## Results

### PRISMA flow chart

**Fig. 1:**
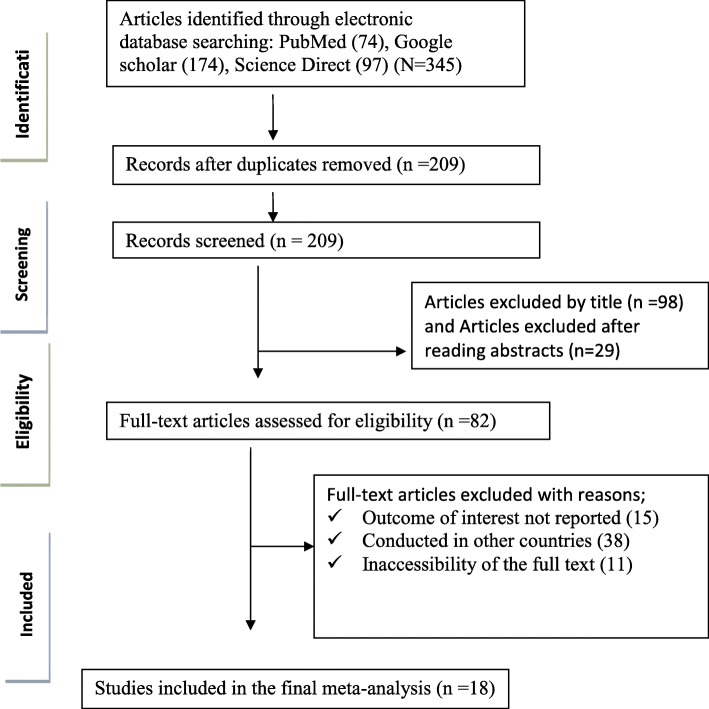
PRISMA study selection flow diagram on first line treatment failure in Ethiopia

We retrieved 345 articles regarding prevalence of treatment failure among ART users as identified in PubMed, Google Scholar, Scopus, and Science Direct. Of these initial articles, 209 articles were non-duplicates; of this, 127 articles were excluded after reviewing their titles and abstracts, and confirmed irrelevance to this review. Thus, 82 potential full text articles were assessed for eligibility, which resulted in further exclusion of 64 articles due to the study conducted in other countries, inaccessibility of the full text articles and outcome of interest not reported. Finally, 18 studies met the eligibility criteria and were included in the final meta-analysis. As shown in the follow chart of study selection process (Fig. 1).

### Characteristics of included studies

As described in Table 1, these 18 studies were both retrospective cohort and cross-sectional study design published prior to March 14, 2019 was included. In the current meta-analysis, 22,849 study participants were represented by 18 studies. The prevalence treatment failure reported was between (4.1%) [17] and (22.2%) [15] and the sample sizes of the studies ranged from 225 [16] to 4809 [21].

**Table 1:** Descriptive summary of 18 studies included in the meta-analysis of the prevalence first line treatment failure in Ethiopia 2019

Authorship	Publication (yr)	Study area /Region	Study population	Study period	Sample	Prevalence at 95% CI
Hail et al. [21]	2016	Oromia	Adult	2007–2014	4809	9.4 (8.6, 10.2)
Ayalew et al. [17]	2016	Amhara	Adult	2011–2015	340	4.1 (2.0, 6.2)
Sisay et al. [20]	2017	Addis Ababa	Adult	2011–2016	595	21.5 (18.2, 24.8)
Melsew et al. [30]	2013	Amhara	Adult	Jan-Apr,2007	509	21.0 (17.5, 24.5)
Teshome et al. [27]	2015	Addis Ababa	Adult	2009–2013	525	19.8 (16.4, 23.2)
Yassin et al. [37]	2017	Oromia	Child	2006–2015	269	18.8 (14.1, 23.5)
Sisay et al. [23]	2017	Amhara	Child	2010–2016	824	7.7 (5.9, 9.5)
Bach et al. [25]	2012	Addis Ababa	Child	2005–2011	1186	14.1 (12.1, 16.1)
Yirdaw et al. [38]	2015	SNNP	Mixed	2004–2012	1321	17.6 (15.6, 19.7)
Assefa et al. [15]	2014	Amhara	Adults	2007–2008	400	22.2 (18.1, 26.3)
Teshome et al. [28]	2015	Addis Ababa	Adult	2007–2009	293	15.7 (11.5, 19.9)
Bekelech et al. [18]	2015	Oromia	Adult	2006–2013	828	6.8 (5.1, 8.5)
Brhane et al. [19]	2017	Amhara	Mixed	Aug-Sep, 2016	421	10.7 (7.8, 13.7)
Zeleke et al. [16]	2016	Amhara	Child	Sept-Dec,2014	225	18.2 (13.2, 23.2)
Agezew et al. [24]	2019	Amhara	Adult	2012–2017	315	10 (6.7,13.3)
Hailu et al. [22]	2018	Tigray	Adult	2008–2016	260	18 (13.3,22.7)
Getaneh et al. [31]	2019	National	Adult	2016–2017	9284	22 (21.2,22.8)
Endebu et al. [26]	2018	Oromia	Adult	2013–2018	445	9 (6.3,11.7)

### Meta-analysis of treatment failure in Ethiopia

**Fig. 2:**
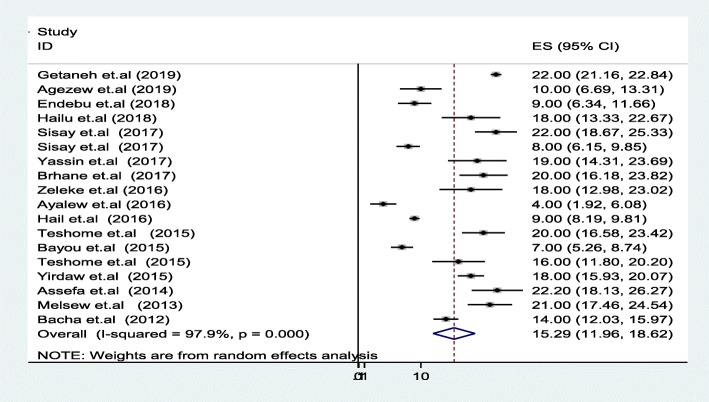
Forest plot of pooled prevalence on first line treatment failure in Ethiopia

Prevalence of ART treatment failure in Ethiopia was 15.3% (95% CI: 12, 18.6) by using the three WHO treatment failure criteria (immunological, clinical and Virological). A high heterogeneity was observed across the included studies (I2 = 97.9%, *p* < 0.001) (Fig. 2). Therefore, a random effect meta-analysis model was computed to estimate the pooled prevalence of treatment failure in Ethiopia. From this meta-analysis, the highest prevalence was 22% (95%CI: 18, 26) reported in a study by Assefa et al. [15], whereas the lowest prevalence of 4% was reported by Ayalew et al. [38]. To identify the possible sources of heterogeneity; different factors associated with it, such as the region of the country where the study was conducted, study population, publication year and sample sizes were investigated by using univariate meta-regression models, but none of these variables were found to be statistically significant (Table 2). We performed an objective based Publication bias assessment using Eggers’ and Beggs’ tests. Accordingly, those tests do not showed statistical significant for prevalence of treatment failure with (*p* = 0.80).

**Table 2:** Related factors with heterogeneity of treatment failure among ART users based on univariate meta-regression

Variables	Coefficient	*P*-value
Publication year	−0.33	0.62
Sample size	0.001	0.3
Region		
Amhara	−3.9	0.57
Addis Ababa	−0.001	0.99
Oromia & SNNP	−6.3	0.4
Study Population		
Adult	0.6	0.9
Child	0.24	0.96

### Subgroup analysis

In this meta-analysis, we computed subgroup analysis based on WHO failure assessment criteria, and the highest prevalence was observed by the immunological failure detection criteria (11.5%) (95%CI: 8.8, 14.3) and the lowest noted by virological confirmation at 5.8% (95% CI: 2.7, 8.9). We also carried out subgroup analysis on the study design yielding a prevalence in cross sectional of 15.9% (95% CI: 9.2, 22.6) nearly the same in retrospective cohort 15.2% (95% CI: 11.5, 18.9). As well, we also considered a subgroup analysis on sample size. Accordingly, the prevalence of first line treatment failure was higher in studies above mean (1269) samples 22% (95% CI: 21.2, 22.8) compared to those with mean sample size below (1269) (15%) (95% CI: 11.8, 18.3).

### Associated factors of ART treatment failure in Ethiopia

**Fig. 3:**
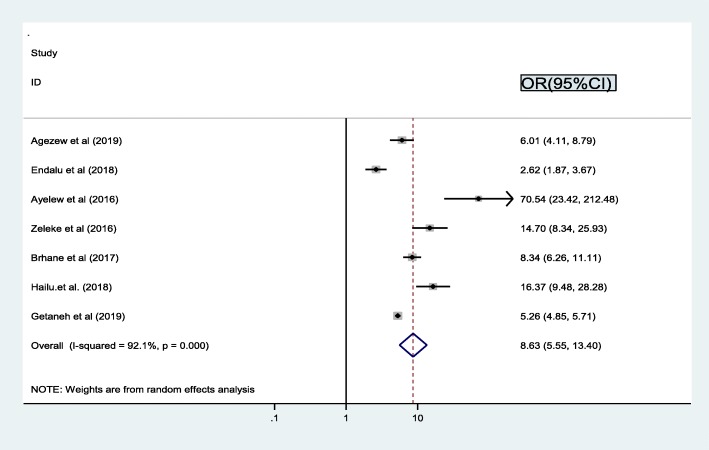
Pooled odd ratio between adherence and treatment failure

In this meta-analysis, we examined the association between ART drug adherence and treatment failure by using seven studies [16, 17, 19, 22, 24, 26, 31]. The findings from these seven studies revealed that the prevalence of treatment failure was significantly associated with adherence. Consequently, the probability of treatment failure was 8.6 times higher among patients who had missed appointments described by greater than 3 days per month (poor adherence) as compared to its counterpart (good adherence) (OR = 8.6, 95% CI: 5.6, 13.4). The result of the statistics indicated that high heterogeneity (I2 = 92.5% and *p* < 0.001) was presented across the included studies (Fig. 3). Accordingly, a random effect meta-analysis model was performed to determine the association.

**Fig. 4:**
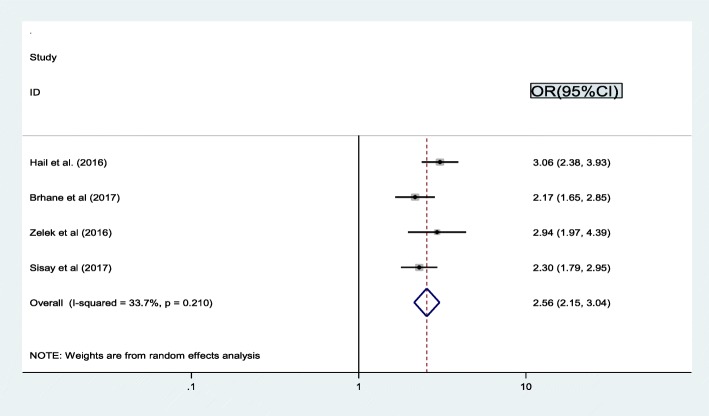
Pooled odd ratio between co-infection and treatment failure

The association between co-infection and treatment failure based on four studies [16, 19–21] showed that the occurrence of treatment failure was associated with co-infection. Subsequently, the probability of treatment failure was 2.6 times higher among patients who had co-infection as compared to patients without co-infection (OR = 2.56, 95% CI: 2.2, 3.0). The statistics indicated moderate heterogeneity (I2 = 33.7% and *p* = 0.210) (Fig. 4). Therefore, a random effect meta-analysis model was executed to determine the association.

**Fig. 5:**
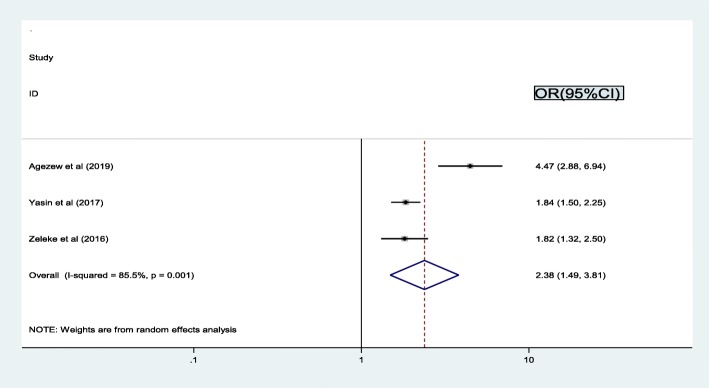
Pooled odd ratio between advanced WHO clinical stage and treatment failure

We observed the association between WHO clinical stage and treatment failure by using three studies [16, 24, 37]. The findings from these three articles revealed that the pooled prevalence of treatment failure was significantly associated with advanced WHO clinical stage. Thus, the likelihood of treatment failure was 2.4 times higher among patients who had advanced WHO clinical stage (III/IV) as compared to stage I and II (OR = 2.4, 95% CI: 1.5, 3.8). The result of the test statistics showed high heterogeneity (I2 = 85.5% and *p* = 0.001) was presented across the articles (Fig. 5). Consequently, a random effect meta-analysis model was computed to determine the association.

**Fig. 6:**
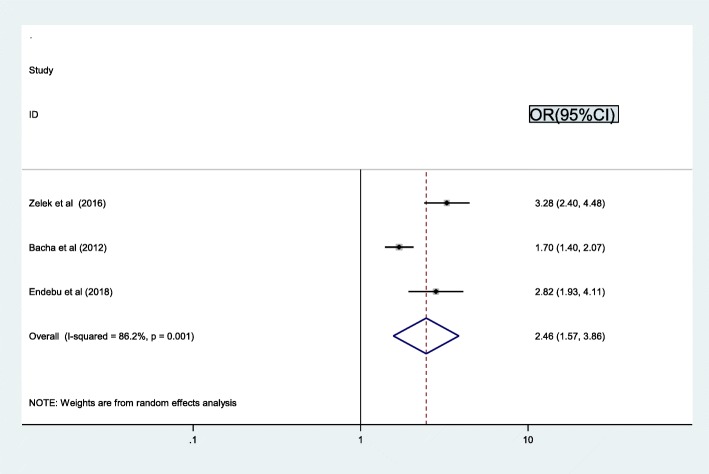
Pooled odd ratio between regimen change and treatment failure

The association between regimen change and treatment failure examined by using three studies [16, 25, 26] revealed the pooled prevalence of treatment failure was associated with regimen change. Therefore, the chance of treatment failure was 2.5 times higher among patients who had regimen change as compared to their counterparts not changing their regimens (OR = 2.5, 95% CI: 1.6, 3.9). The result of test statistics indicated that high heterogeneity (I2 = 86.2% and *p* = 0.001) (Fig. 6). Hence, a random effect meta-analysis model was used to determine the association with the outcome.

**Fig. 7:**
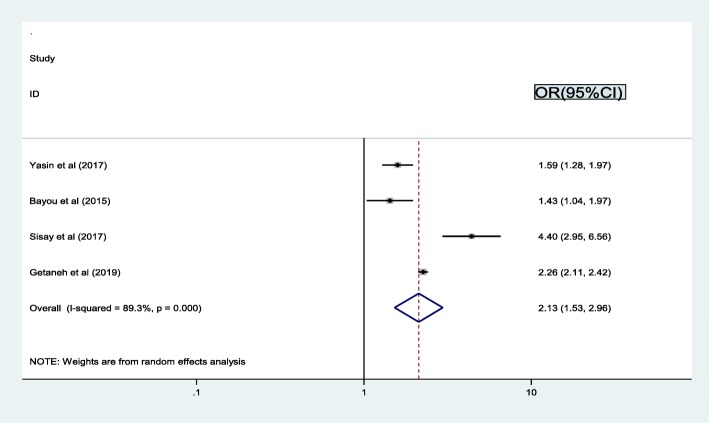
Pooled odd ratio between disclosure and treatment failure

Similarly, the association between discloser and treatment failure based on four studies [18, 23, 31, 37] showed that the prevalence of treatment failure was associated with disclosure. Therefore, the possibility of treatment failure was 2 times higher among patients who had not disclosed as compared to disclosed (OR = 2.1, 95% CI: 1.5, 3.0). The result of test statistics indicated high heterogeneity (I2 = 89.3% and *p* < 0.001) (Fig. 7). Therefore, a random effect meta-analysis model was used to determine the association with the outcome.

Finally, we examined the association between treatment failure and CD4 count as well as functional status with six and three studies respectively. However, both of these factors were not statistically associated with treatment failure.

## Discussion

Treatment failure is one of the causes of mortality and development of drug resistant viral strains potentiating a significant challenge globally [10, 11]. To the best of our knowledge, this meta-analysis is the first of its kind to estimate the pooled prevalence of treatment failure in Ethiopia.

The overall prevalence of first line ART treatment failure identified in this study showed that 15.3% (95% CI: 12, 18.6) ART user patients faced first line treatment failure in Ethiopia. The result of this meta-analysis is in line with that of a study conducted in Haiti (15%) [39], higher than that of studies in Burkina Faso (6.4%), Ghana (6.5%), and Tanzania (7%) [40–42] and lower than the result in Uganda (34%) [43]. The possible explanation for the observed variations could be attributed to methodological difference in the assessment of failure, sample size, socio-economic, and medical service that has a great impact on treatment failure detection.

We did subgroup analysis due to a significant heterogeneity as shown in (Fig. 2) which indicated the highest prevalence of treatment failure was immunological (11.5%), followed by clinical failure (6.7%), whereas the lowest was virological (5.8%). The reason behind is clinical and immunological criteria were found to perform relatively poor in predicting virological failure of ART [44]. The possible explanation for this variation could be that immunological and clinical criteria have poor sensitivity and positive predictive value to detect treatment failure, particularly immunological failure relying on higher CD4 cell counts for treatment monitoring would therefore lead to misclassifications of treatment failure [45–48]. In addition, regular virological treatment failure detection is low because of inadequate capacity and laboratory facilities in Ethiopia.

Likewise, the subgroup analysis of this study on mean sample size with below and above the mean but both indicated almost equal to the overall pooled prevalence with 15 and 16.3% respectively. The possible explanations for this similarity could be due to the mean samples size are sufficiently large. Consequently, the larger sample increases the estimation of parameters both above and below mean.

The region of the country, type of failure assessment criteria, study population, publication year and sample sizes were investigated by using univariate meta-regression models, but none of these variables were found to be statistically significant. Publication bias using Egger’s tests did not show statistical significant for estimating the prevalence of treatment failure among ART users with *p* = 0.80.

This meta-analysis also aims to identify the possible determinant factors on the magnitude of treatment failure among HIV patients in Ethiopia. In this study, adherence, co-infection, advanced WHO clinical stage, disclosure, and regimen change were found statistically associated factors for treatment failure. The success of ART reflects, in part, patient adherence to treatment and the present finding revealed that adherence is significantly indication of treatment failure [49]. Accordingly, the probability of treatment failure was 8.6 times higher among patients who did not use ART continuously Poor adherence is one of the ways in which drug adaptability and resistance developed [50].

Disclosure is another determinant factor for treatment failure. In line with study in Tanzania, this study showed that not disclose HIV infection status was two times higher risk of treatment failure [50]. Basically non-disclosure leads to ‘hidden behaviors’ and potentiates non-adherence as a cause for treatment failure. However, one study conducted Ethiopia, showed non-disclosure as a protective factor for treatment failure due to stigma and discrimination [17].

In addition, co-infection is an associated factor of treatment failure among patients on ART. As supported by studies in South Africa and Uganda, co-infection was a determinant factor for ART treatment failure [51, 52]. This could be due to having advanced opportunistic infection/co-infection which may deplete CD4 counts and compromise immunity and may negatively affect response to treatment. In addition, medications for co-infection treatment and ART together can contribute to double burden side effects.

Pooled prevalence of treatment failure was associated with regimen change. Regimen change yield a 2.5 times higher possibility of developing treatment failure which is similar with studies conducted in Myanmar and Malawi [53, 54].

Finally, advanced WHO clinical stage was a significant predictor of ART treatment failure as indicated in studies in South Africa and Uganda [55, 56]. The likelihood of treatment failure was 2.4 times higher among patients in advanced clinical stage III/IV as compared to stage I/II in this meta-analysis. This finding could be due to advanced HIV disease which is often associated with loss to follow-up [57].

Even though the study is nationally based; including only English articles and having a relatively small sample size was the limitation of the study. In addition this meta-analysis represented only studies reported from three regions and two administrative town of the country, which could bias the estimate of treatment failure proportion for the entire Ethiopian context. Furthermore, the included studies did not incorporated a number of factors such as age, sex, duration of follow up, opportunistic infection, and body mass index as a possible factor to examine the pooled odds ratios.

## Conclusion

In this study, treatment failure among ART users in Ethiopia was significantly high. Adherence, co-infection, advanced WHO clinical stage, regimen change and disclosure are determinant factors for treatment failure. Therefore, improved drug adherence, prevention of co-infection, as well as timely and rigorous follow up, were found to sustain first-line therapy effectiveness and prevent HIV-drug resistance.

## Data Availability

Minimal data could be accessed upon request from first author (MAA).

## Abbreviations

AIDS: Acquired immune deficiency syndrome
ART: Antiretroviral therapy
HAART: Highly active antiretroviral therapy
HIV: Human immune virus
PRISMA: Preferred Reporting Items for Systematic Reviews and Meta-Analysis
WHO: World Health Organization
